# Development and External Validation of an Efficient Deep Learning Model for Lesion Segmentation and Subtyping of Hepatic Echinococcosis in Ultrasound Imaging

**DOI:** 10.1002/hcs2.70077

**Published:** 2026-05-19

**Authors:** Zhu He, Jiajun Qiu, Chenlin Du, Jin Yin, Xuhui Zhang, Yelei Ren, Yifei Wang, Lamu Suolang, Chunyang Li, Zongjiu Zhang, Diming Cai, Qicheng Lao

**Affiliations:** ^1^ School of Artificial Intelligence, Beijing University of Posts and Telecommunications Beijing China; ^2^ Department of Medical Ultrasound and West China Biomedical Big Data Center West China Hospital, Sichuan University Chengdu China; ^3^ Med‐X Center for Informatics, Sichuan University Chengdu China; ^4^ Department of Geriatric Dentistry Peking University School and Hospital of Stomatology & National Center for Stomatology & National Clinical Research Center for Oral Diseases & National Engineering Research Center of Oral Biomaterials and Digital Medical Devices Beijing China; ^5^ Department of Medical Ultrasound West China Hospital, Sichuan University Chengdu China; ^6^ Center of Disease Control and Prevention, Xizang Autonomous Region Lhasa China; ^7^ Institute for Hospital Management, Tsinghua Medicine, Tsinghua University Beijing China; ^8^ Shanghai Artificial Intelligence Laboratory Shanghai China

**Keywords:** classification, deep learning, hepatic echinococcosis, segmentation, ultrasound image

## Abstract

**Background:**

Hepatic echinococcosis is a zoonotic parasitic disease common in remote, resource‐limited pastoral regions. It mainly appears as cystic echinococcosis (CE), due to Echinococcus granulosus, and alveolar echinococcosis (AE), caused by multilocular Echinococcus species. CE can lead to biliary colic or vascular compression, while AE often mimics liver cancer and exhibits high long‐term mortality. Although ultrasonography is the diagnostic method of choice, its accuracy is affected by imaging artifacts and operator variability. Conventional deep learning models are often computationally heavy and lack interpretability, limiting their use in underdeveloped areas. Integrating frequency‐ and spatial‐domain features in a lightweight framework offers a promising solution for efficient segmentation and classification of hepatic echinococcosis. The aim of this study is to develop efficient deep learning models for hepatic echinococcosis segmentation and classification, facilitating large‐scale screening with non‐invasive, portable ultrasound imaging.

**Methods:**

This study utilized a large ultrasound dataset to train and evaluate a deep learning model, consisting of 20,112 images from 4437 patients in Shiqu County, Sichuan Province, China, an endemic area for hepatic echinococcosis. To further assess the model's robustness, an external dataset comprising 3340 images from 1123 patients at West China Hospital of Sichuan University was used for additional testing. By enhancing the correlation of image features in both the frequency and spatial domains for hepatic echinococcosis ultrasound images, and incorporating segmentation features to assist the classification task, the developed model achieves high efficiency and lightness.

**Results:**

The proposed model achieved a Dice coefficient of 80.67% and 78.12% for segmentation, and classification accuracy of 90.10% and 80.96% on the internal and external test sets, respectively. Compared to the lightweight state‐of‐the‐art (SOTA) model, it improves inference speed by 43.48% and increases classification accuracy by 8.89% and 16.32% on internal and external test sets, respectively. Compared to the standard SOTA model, it is only 8% of its size but boosts inference speed by 821.37%, with classification accuracy improvements of 3.65% and 4.57% on internal and external test sets, respectively.

**Conclusions:**

The proposed model offers efficient and accurate hepatic echinococcosis diagnosis, with a lightweight design suitable for both resource‐limited and advanced clinical settings.

## Graphical abstract

We developed a lightweight deep learning framework that integrates frequency‐ and spatial‐domain ultrasound features with segmentation‐assisted classification for hepatic echinococcosis diagnosis. The model outperformed existing state‐of‐the‐art methods in both accuracy and inference speed while requiring substantially fewer computational resources. Its efficient and compact design makes it well‐suited for large‐scale ultrasound screening in resource‐limited endemic regions.

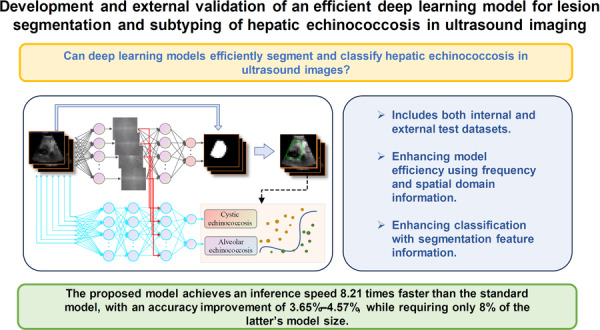

**Abbreviations**
Acc.: accuracy
AE: alveolar echinococcosis
AUC: area under the ROC curve
CT: computed tomography
CE: cystic echinococcosis
DGA: deformable global attention
Dice: dice similarity coefficient
FDA: frequency domain attention
FEP: frequency excitation and pruning
FLOPs: floating‐point operations
FPS: frames per second
HD95: 95% Hausdorff distance
IoU: intersection over union
ROC: receiver operating characteristic
SOTA: state‐of‐the‐art

## 1 Background

**Figure 1:**
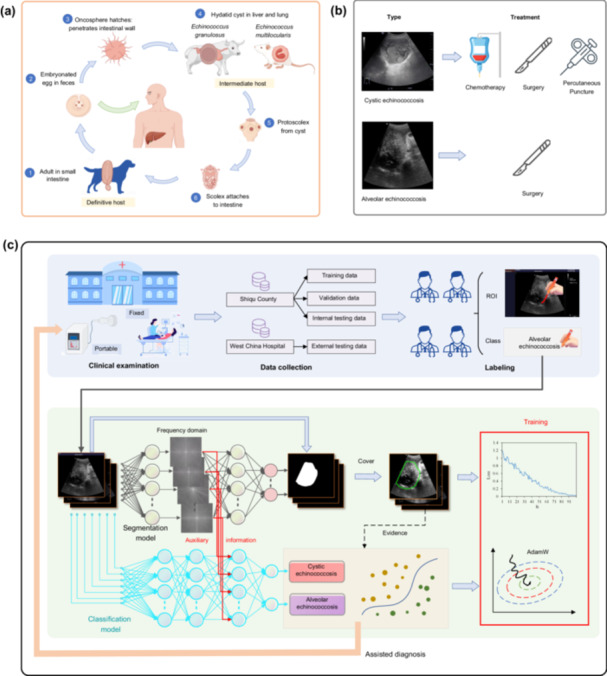
Overview of hepatic echinococcosis and the proposed approach. (a) The infection process of hepatic echinococcosis. (b) The treatment of the two different subtypes of cystic echinococcosis and alveolar echinococcosis. (c) The concept of the proposed approach.

Hepatic echinococcosis is a common zoonotic parasitic disease caused by infection with the Echinococcus tapeworm. It is prevalent in pastoral areas and is widely distributed in Africa, South America, Europe, and Asia [1]. This disease presents primarily in two forms: cystic echinococcosis (CE) and alveolar echinococcosis (AE) [2]. CE results from infection with the larvae of Echinococcus granulosus, while AE is caused by infection with multilocular Echinococcus species. The pathophysiology of these two forms varies significantly (Figure 1a). CE cysts may rupture into the biliary tree, causing biliary colic, or exert pressure on the portal vein, hepatic vein, or inferior vena cava, leading to portal hypertension or venous obstruction. AE can cause liver enlargement, with clinical manifestations resembling liver cancer. The mortality rate for patients with CE is approximately 2%–4%, whereas the 10‐ to 15‐year mortality rate for patients with AE can be as high as 90% [3]. The treatment approaches for these two types of infection differ significantly (Figure 1b), where the treatment methods of CE mainly include surgery, chemotherapy, and percutaneous puncture, while surgery is the preferred treatment for AE, and co‐infection of two pathogens is rare [4]. Therefore, accurate classification of hepatic echinococcosis is crucial for precise treatment and improving patient prognosis.

Ultrasonography is a widely employed non‐invasive diagnostic modality in medical practice and is considered the preferred method for diagnosing hepatic echinococcosis [5]. Compared to computed tomography (CT), a commonly utilized alternative imaging technique for hepatic echinococcosis screening, ultrasonography presents significant advantages in terms of cost‐effectiveness and accessibility, rendering it particularly suitable for screening programs in resource‐limited and endemic areas. However, the imaging quality of ultrasonography can be influenced by factors such as patient body habitus, and interpretation is dependent on operator expertise [6]. To address these inherent limitations, the application of deep learning‐based automatic segmentation and classification of hepatic echinococcosis lesions is being actively investigated to assist clinicians in diagnosis, with the aim of reducing diagnostic errors and missed diagnoses, thereby ultimately improving diagnostic efficiency [7, 8].

However, several challenges remain to be addressed in aiding the diagnosis of hepatic echinococcosis. First, during ultrasound imaging, the propagation of sound waves through biological tissues can introduce artifacts, and tissue heterogeneity can further distort the waves, making image interpretation more complex [9]. Second, conventional deep learning classification models typically extract features implicitly and directly output classification predictions, which limits their interpretability—an essential requirement for hepatic echinococcosis diagnosis [10]. Finally, screening for hepatic echinococcosis predominantly occurs in underdeveloped regions, where access to advanced computational resources is often limited. Many existing diagnostic models require substantial computational power, making them unsuitable for deployment in resource‐constrained clinical settings, particularly on portable devices [11]. Developing lightweight, efficient models capable of operating under these constraints remains a pressing need [12]. Additionally, while frequency‐domain analysis has shown promise in ultrasound imaging tasks, its potential for hepatic echinococcosis diagnosis remains underexplored [13]. Leveraging frequency‐domain features alongside advanced techniques like self‐attention mechanisms could enhance diagnostic accuracy while maintaining computational efficiency.

Therefore, this study introduces a novel method that integrates both frequency‐domain and spatial‐domain features for the segmentation and classification of hepatic echinococcosis. Specifically, the proposed model leverages frequency‐domain information to compensate for blurred spatial‐domain features, enhancing the perceptibility of ultrasound images of echinococcosis. Additionally, the model performs a segmentation task and utilizes the feature maps from the segmentation model to assist in hepatic echinococcosis classification, thereby improving interpretability—a crucial aspect in medical diagnosis. To address computational constraints, a lightweight design was implemented, ensuring the model requires minimal computational resources while maintaining excellent segmentation and classification performance. The robustness of the proposed model was further validated using an external test set, confirming its effectiveness and reliability in diverse settings. Our model is adaptable for use on imaging devices in both primary healthcare settings (Shiqu County, Garze Tibetan Autonomous Prefecture, Sichuan Province, China) and tertiary hospital (West China Hospital, Sichuan University).

Deep learning has become a mainstream approach for automated diagnosis of hepatic echinococcosis due to its high accuracy and efficiency [14]. Previous studies have applied CNN‐based models for classification and segmentation tasks. For example, Wu et al. [15] used VGG19, Inception‐v3, and ResNet18 to classify hepatic cystic echinococcosis subtypes, with VGG19 achieving 90.6% accuracy. Xin et al. [16] developed networks for CT‐based segmentation and classification, and Huang et al. [17] employed U‐Net models for ultrasound segmentation. Several studies have explored AI‐assisted diagnostic systems using large ultrasound datasets. Yang et al. [18] created a dataset of 9631 images and evaluated multiple deep learning models, while Wang et al. [19] developed EDAM to differentiate hepatic encapsulated disease from liver cysts and normal tissue. Using segmentation outputs to aid classification enhances focus on relevant regions and improves interpretability. However, previous methods, such as sequential segmentation‐classification [16] or shared backbone [17] approaches, are prone to error accumulation or gradient normalization challenges [20]. In our study, we leverage the intermediate feature maps generated by the segmentation model to enhance the classification task, aiming to offer a more robust auxiliary diagnostic solution for hepatic echinococcosis.

Transformer‐based models have recently demonstrated strong potential in medical image segmentation. By leveraging self‐attention mechanisms [21], Transformers overcome the limited receptive field of convolutional networks, enabling long‐range dependency modeling and improved global feature representation. However, the quadratic computational complexity of self‐attention [22] significantly increases the computational burden when multiple Transformer modules are used. To address this, Lin et al. [23] introduced an adaptive pruning strategy to remove redundant computations in Transformer‐based segmentation models, effectively reducing model size. Nevertheless, this approach requires learning additional pruning parameters during training, which increases training complexity and slows inference. Given these challenges, developing more efficient and lightweight Transformer‐based models remains crucial, and it is particularly important for accurate and practical ultrasound detection of hepatic echinococcosis.

Some studies [24, 25] have shown that frequency‐domain networks can serve as an alternative to self‐attention for global feature modeling, offering linear computational complexity and faster inference. Prior studies have explored using frequency‐domain operations to reduce the cost of self‐attention. Global filtering [26] and Fourier‐based kernels [27] have been shown to model long‐range dependencies more efficiently and with less redundancy. Hybrid approaches that mix spectral modules with self‐attention [28] further improve feature representation, though the relationship between the two domains is not explicitly modeled. Motivated by these findings, we design a frequency‐guided excitation and pruning strategy that adaptively enhances self‐attention on features with important frequency information and suppresses less relevant ones. This coupled use of frequency‐ and spatial‐domain cues supports more effective segmentation and classification of hepatic echinococcosis in ultrasound images.

## 2 Methods

### 2.1 Ethics Statement

The study protocol was approved by the Ethics Committee of West China Hospital, Sichuan University (Approval 2019482). Due to the retrospective nature of the study, the requirement for written informed consent was waived.

### 2.2 Overview of the Study

The coupling frequency‐ and spatial‐domain contextual dependencies network (CopFSNet) is designed for the segmentation and recognition of hepatic echinococcosis in ultrasonic images (Figure 1c). CopFSNet operates by integrating contextual dependencies from both frequency and spatial domains, enabling precise segmentation of hepatic echinococcosis lesions. The model was developed using an ultrasonic dataset comprising 20,112 images collected from 4437 patients in Shiqu County, Garze Tibetan Autonomous Prefecture, Sichuan Province, China, a high‐burden area for hepatic echinococcosis. To further validate the robustness of the proposed CopFSNet, an external dataset of 3340 images from 1123 patients was collected from a tertiary hospital (West China Hospital, Sichuan University) for additional testing.

### 2.3 Patient Enrollment

In this retrospective study, a total of 4437 patients from Shiqu County, Garze Tibetan Autonomous Prefecture, Sichuan Province, China (a high‐altitude epidemic area), diagnosed between October 2014 and October 2019, were retrospectively included for the development of the deep learning model. For external validation, an additional 1,123 patients diagnosed at West China Hospital, Sichuan University (a tertiary hospital in a major city), between April 2005 and December 2022, were enrolled. Diagnoses of AE or CE were confirmed by two senior ultrasonic radiologists with consensus, each with over 6 years of expertise in abdominal ultrasound diagnosis.

Inclusion criteria required patients to display typical sonographic features of AE or CE in either gray‐scale or color Doppler images, with no detectable blood flow within the lesions. Patients were excluded if their sonograms were non‐diagnostic (e.g., absent or atypical lesions), if color Doppler imaging revealed blood flow within the lesions, or if images were of low quality or contained artifacts such as markers, measurement lines, or other visual obstructions that could interfere with computational analysis.

### 2.4 CopFSNet Development

**Figure 2:**
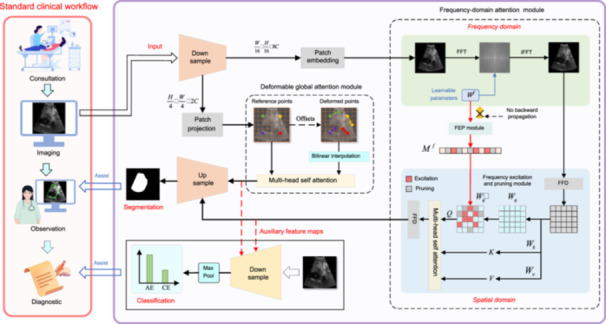
The framework of our proposed CopFSNet model. This model mainly consists of the frequency‐domain attention (FDA) module, the frequency excitation and pruning (FEP) module, and the deformable global attention (DGA) module for segmentation and classification of hepatic echinococcosis in ultrasound images. AE, alveolar echinococcosis; CE, cystic echinococcosis; DGA, deformable global attention; FDA, frequency domain attention; FEP, frequency excitation and pruning.

As illustrated by Figure 2, CopFSNet incorporates three lightweight modules to improve performance: Frequency Domain Attention (FDA), Frequency Excitation and Pruning (FEP), and Deformable Global Attention (DGA). FDA models contextual dependencies in both frequency and spatial domains using frequency‐domain transformations and self‐attention mechanisms. FEP enhances spatial‐domain self‐attention with excitation methods, prioritizing important features in the frequency domain and reducing redundancy through pruning. DGA constructs multi‐level spatial features, improving feature extraction in ultrasound images. These modules reduce redundant attention on irrelevant areas, focusing on the lesion region to improve segmentation accuracy. A detailed explanation of the three modules, including their theoretical formulations, as well as the training and optimization details of the CopFSNet model, is provided in the Supporting Materials.

Note that the CopFSNet handles segmentation and classification tasks separately. For segmentation, a U‐shaped symmetric encoder‐decoder architecture is used, with the FDA module applied at 16× downsampling and DGA at 4× downsampling to ensure efficiency. The binary cross‐entropy and Dice loss functions are used for calculating segmentation loss. For classification, features from the segmentation encoder assist in the task, with a cross‐entropy loss function used to train the classification model, which helps in determining the presence of hepatic echinococcosis.

### 2.5 Patient Level Classification

Given that the ultimate goal of clinical diagnosis is to provide a definitive diagnosis for the patient, we further develop a clinical decision support system that integrates CopFSNet to achieve patient‐level diagnosis [29, 30]. Specifically, the patient‐level diagnosis is determined by employing a voting method based on the classification results of each individual image. The patient's predicted category is determined by the majority classification result from all images. In the case of a tie, the class with the highest average predicted probability is selected.

### 2.6 Evaluation and Statistical Analysis

During the training phase, the model's performance is validated on the validation set at the end of each epoch, and the model parameters yielding the best performance on the validation set are selected. The model's final performance is evaluated on both the internal and external test sets. We evaluate the segmentation performance of the model using four metrics: dice similarity coefficient (Dice), Intersection over Union (IoU), ACC, and 95% Hausdorff distance (HD95). The segmentation results of our proposed CopFSNet model are then compared with those of several standard models (U‐Net [31], Att‐UNet [32], Swin‐UNet [33], FAT‐Net [34], ACC‐UNet [35], CFATrans [36], TBConvL‐Net [37]) as well as lightweight models, such as LM‐Net [38], MISSFormer [39], FF‐UNet [40], Ms RED [41], the model proposed by Zhu et al. [42] and MBF‐Net [43].

For the classification task, we employ metrics including accuracy (Acc.), precision, recall, F1‐score, and Receiver Operating Characteristic (ROC) curves, along with the area under the ROC curve (AUC) for comparison. We compare our proposed method, CopFSNet, with several standard architectures (ResNet50 [44], ConvNeXt [45], InceptionV3 [46], ConvViT [47], HFFDL [48]) and other lightweight models (MobileNetV2 [49], GhostNet [50], ShuffleNetV2 [51], RepVit [52], EL‐CNN [53]) to validate its performance.

Additionally, for both segmentation and classification tasks, we report the parameter count (Params) and the required number of floating‐point operations (FLOPs) for each model to compare their sizes, as well as the frames per second (FPS) to assess inference speed.

## 3 Results

### 3.1 Dataset Characteristics

**Figure 3:**
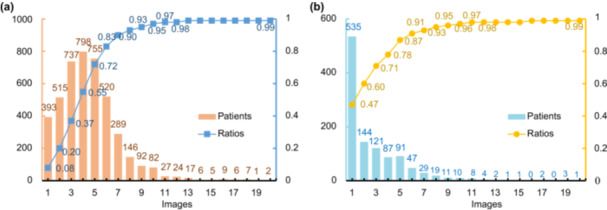
Patient statistics with varying numbers of images (no more than 20) in the datasets: (a) internal dataset; (b) external dataset.

As shown in Figure 3a, 90% of patients in the internal dataset had no more than eight images, with only six patients having more than 20 images. However, 47% of the patients in the external validation dataset have only a single image (Figure 3b), with only seven patients having more than 20 images, making it highly suitable for validating the model's generalization ability. Therefore, this dataset was used as an external test set. Detailed statistics regarding the distribution and quantity of images and patients in both datasets are provided in Table 1. We provide detailed information on the ultrasound devices, probes, acquisition protocols, and image post‐processing procedures, as well as stratified statistics for CE and AE cases, including lesion size distribution, centroid location distribution, and shape characteristics, in the Supporting Materials.

**Table 1:** Numbers of images and patients in the training set, validation set, internal test set, and external test set used in this study.

Variables	Internal set			External set
	Training	Validation	Internal testing	External testing
CE	5632	704	705	7041
AE	10,468	1297	1306	13,071
Total	16,100	2001	2011	20,112
$N_{\mathrm{CE}}$	1240	155	240	1637
$N_{\mathrm{AE}}$	2310	287	205	2800
$N_{\mathrm{total}}$	3550	442	445	4437

*Note: N* indicates the number of patients.

Abbreviations: AE, alveolar echinococcosis; CE, cystic echinococcosis.

### 3.2 Segmentation Performance

Tables 2 and 3 demonstrate that CopFSNet, which integrates FDA, FEP, and DGA modules, outperformed all compared models, achieving a Dice coefficient of 80.67% on the internal validation dataset and 78.12% on the external validation dataset. Additionally, it delivered superior performance across five other segmentation evaluation metrics, demonstrating its effectiveness, robustness, and generalizability.

**Table 2:** Segmentation performance and model size comparison of deep learning models on the internal test set.

Internal test	Dice	IoU‐B	IoU‐F	Acc.‐B	Acc.‐F	GAcc.	Params↓	GFLOPs↓	FPS↑	HD95↓
Standard model										
U‐Net	0.7916	0.9752	0.7267	0.9871	0.8417	0.9767	31.04	65.31	89.70	14.83
Att‐Unet	0.7853	0.9707	0.7246	0.9822	0.8405	0.9653	33.74	64.52	83.21	15.38
Swin‐UNet	0.7686	0.9684	0.7018	0.9768	0.8289	0.9405	42.46	27.17	43.62	18.24
FAT‐Net	0.7994	0.9767	0.7391	0.9884	0.8483	0.9782	28.76	30.53	27.54	13.85
ACC‐UNe	0.7942	0.9753	0.7312	0.9856	0.8425	0.9737	16.82	38.34	14.61	14.17
CFATrans	0.8012	0.9784	0.7395	0.9902	0.8496	0.9796	66.06	42.48	11.59	13.36
MBF‐Net	0.7923	0.9748	0.7307	0.9838	0.8421	0.9720	32.47	34.68	35.75	14.29
Lightweight model										
LM‐Net	0.7714	0.9706	0.7158	0.9806	0.8286	0.9628	5.43	**4.68**	61.79	16.04
MISSFormer	0.7846	0.9742	0.7204	0.9865	0.8382	0.9729	5.88	7.21	25.94	15.60
FF‐UNet	0.7724	0.9716	0.7149	0.9814	0.8256	0.9682	**3.94**	10.43	97.25	15.83
Ms RED	0.7698	0.9692	0.6943	0.9785	0.8213	0.9438	4.70	9.00	36.42	18.86
Zhu et al. [42]	0.7888	0.9743	0.7182	0.9881	0.8239	0.9759	4.28	38.58	28.54	15.18
TBConvL‐Net	0.7906	0.9754	0.7208	0.9893	0.8396	0.9772	9.62	15.54	57.63	14.76
CopFSNet	**0.8067**	**0.9789**	**0.7411**	**0.9912**	**0.8506**	**0.9812**	4.03	6.39	**115.83**	**12.86**

*Note:* “B” and “F” denote foreground (lesion areas) and background (other areas), respectively. “GAcc.” represents the global accuracy. The unit of the number of model parameters (Params) is million (M). 1 GFLOPs is equal to 10e9 floating‐point operations (FLOPs). “↓” indicates that smaller values are better, and “↑” indicates the opposite. The bold values in the table indicate the best results.

Abbreviations: Acc., accuracy; Dice, dice similarity coefficient; FPS, frames per second; FLOPs, floating‐point operations; HD95, 95% Hausdorff distance; IoU, intersection over union.

**Table 3:** Segmentation performance and model size comparison of deep learning models on the external test set.

External test	Dice	IoU‐B	IoU‐F	Acc.‐B	Acc.‐F	Gacc.	HD95↓
Standard model							
U‐Net	0.7492	0.9496	0.6512	0.9732	0.8001	0.9438	18.42
Att‐Unet	0.7567	0.9487	0.6568	0.9711	0.8096	0.9533	17.65
Swin‐UNet	0.7351	0.9339	0.6329	0.9560	0.7879	0.9324	21.78
FAT‐Net	0.7624	0.9484	0.6585	0.9695	0.8204	0.9530	17.82
ACC‐UNe	0.7585	0.9443	0.6498	0.9623	0.8166	0.9496	18.21
CFATrans	0.7669	0.9502	0.6584	0.9741	0.8279	0.9546	17.57
MBF‐Net	0.7647	0.9493	0.6587	0.9708	0.8209	0.9537	17.60
Lightweight model							
LM‐Net	0.7435	0.9459	0.6443	0.9687	0.8063	0.9507	19.88
MISSFormer	0.7483	0.9457	0.6483	0.9627	0.8072	0.9383	19.32
FF‐Unet	0.7382	0.9421	0.6384	0.9635	0.7964	0.9482	21.03
Ms RED	0.7487	0.9484	0.6527	0.9719	0.8073	0.9529	19.32
Zhu et al. [42]	0.7426	0.9421	0.6470	0.9604	0.7918	0.9368	19.60
TBConvL‐Net	0.7517	0.9495	0.6525	0.9723	0.8070	0.9538	18.47
CopFSNet	**0.7812**	**0.9527**	**0.6634**	**0.9782**	**0.8327**	**0.9604**	**15.64**

*Note:* “B” and “F” denote foreground (lesion areas) and background (other areas), respectively. “GAcc.” indicates global accuracy. “↓” indicates that smaller values are better. The bold values in the table indicate the best results.

Abbreviations: Acc., accuracy; Dice, dice similarity coefficient; HD95, 95% Hausdorff distance; IoU, intersection over union.

**Figure 4:**
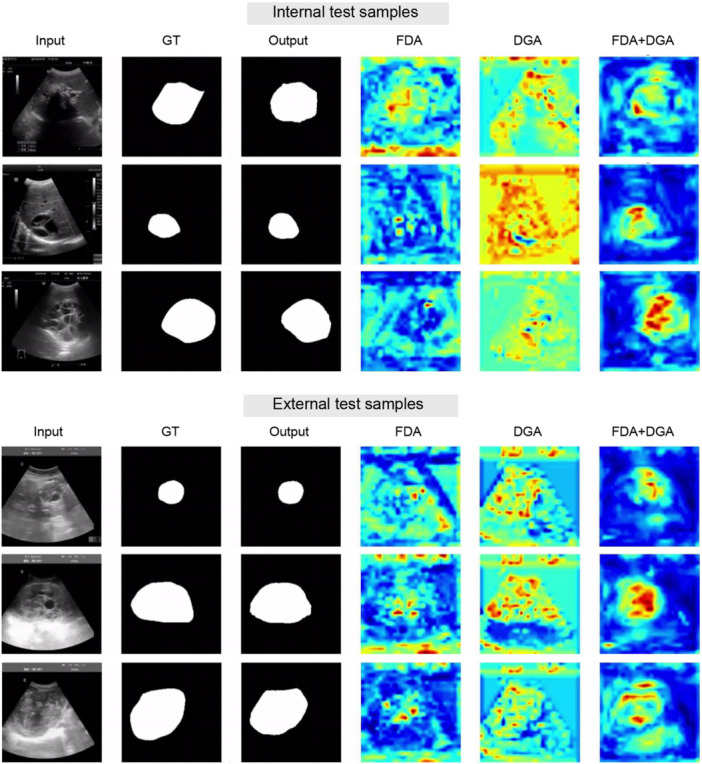
Visualization of the segmentation results and the feature heatmaps of the CopFSNet. From left to right are the original input images, the ground truth (GT), the predicted segmentation results of the CopFSNet, the feature heatmaps of the FDA module, the feature heatmaps of the DGA module, and the heatmaps after fusion of the features in the FDA and DGA modules. DGA, deformable global attention; FDA, frequency domain attention; GT, ground truth.

Figure 4 displays the segmentation output of the CopFSNet model along with the feature heatmaps from the model's internal modules, FDA and DGA. The FDA module, designed for frequency‐domain feature extraction, shows strong lesion localization, focusing on key features within the lesions. However, it does not fully capture the entire lesion regions, as it is located after the 16× convolutional downsampling in the CopFSNet model, where the frequency‐domain transformation emphasizes finer image details. In contrast, the DGA module, located after the 4× convolutional downsampling layer and modeled entirely using self‐attention, provides broader global attention. The heatmaps in the “FDA + DGA” column, which combine the outputs from both modules, are more coherent and effective compared to those generated by the FDA or DGA modules individually. This indicates that the CopFSNet model effectively merges information from both modules to achieve features with improved localization and a broader perceptual range.

### 3.3 Image Level Classification Performance

**Figure 5:**
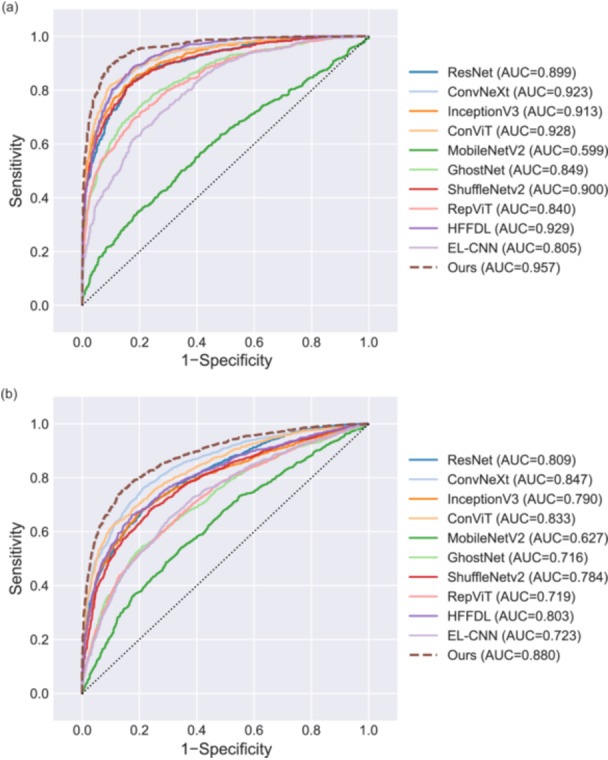
Comparison of ROC curves and area under the curve for the image‐level classification task: (a) internal test set; (b) external test set. AUC, area under the ROC curve; ROC, receiver operating characteristic.

As shown in Table 4, the proposed model improves the classification accuracy by 7.16% on the internal test set and 7.31% on the external test set compared to the ResNet50 model. Figure 5 illustrates the classification ROC curves and AUC for our proposed CopFSNet model and other models on both the internal and external test sets. Our model achieves the highest AUC values of 0.957 and 0.880 on the internal and external test sets, respectively, demonstrating its superior performance.

**Table 4:** Classification performance and model size comparison of deep learning models on the internal and external test set.

Models	Internal test				External test				Params	GFLOPs	FPS
	Acc.	Precsion	Recall	F1‐score	Acc.	Precision	Recall	F1‐score			
Standard model											
ResNet50	0.8294	0.7633	0.7382	0.7505	0.7365	0.7187	0.6893	0.7037	23.51	4.13	44.33
ConvNeXt	0.8593	0.8059	0.7840	0.7948	0.7651	0.7732	0.7028	0.7342	29.00	4.50	37.45
InceptionV3	0.8473	0.7865	0.7697	0.7780	0.7308	0.7015	0.7084	0.7050	21.79	2.85	23.73
ConViT	0.8672	0.8439	0.7582	0.7988	0.7563	0.7675	0.6642	0.7122	27.00	5.40	32.38
HFFDL	0.8675	0.8428	0.7595	0.7994	0.7360	0.7194	0.6887	0.7034	38.47	6.78	24.03
Lightweight model											
MobileNetV2	0.6738	0.6126	0.1674	0.2629	0.5994	0.6440	0.2625	0.3730	4.13	0.61	62.20
GhostNet	0.7966	0.7535	0.6166	0.6782	0.6581	0.6190	0.6418	0.6302	4.28	**0.25**	41.58
ShuffleNetV2	0.8359	0.7733	0.7468	0.7598	0.7132	0.6744	0.7117	0.6926	3.50	1.10	69.25
RepViT	0.7882	0.7571	0.5751	0.6537	0.6659	0.6414	0.5983	0.6191	6.80	2.20	58.43
EL‐CNN	0.7445	0.7432	0.5417	0.6015	0.6721	0.6512	0.6437	0.6688	3.72	2.88	62.71
CopFSNet	**0.9010**	**0.8592**	**0.8555**	**0.8573**	**0.8096**	**0.8284**	**0.7321**	**0.7773**	**3.42**	0.67	**85.35**

*Note:* “Acc.” indicates classification accuracy. “ResNet50_GT” indicates that the images containing only the lesion region are used as input to the ResNet50 model. The bold values in the table indicate the best results.

Abbreviations: Acc., accuracy; FPS, frames per second; FLOPs, floating‐point operations.

### 3.4 Patient Level Classification Performance

**Figure 6:**
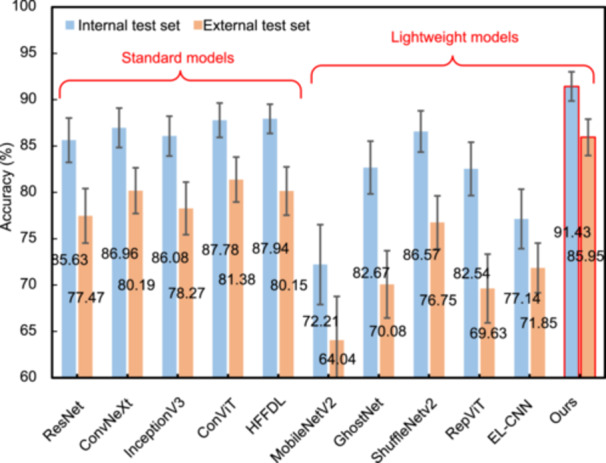
Comparison of accuracy and prediction bias in internal and external test sets on patient‐level classification tasks.

Figure 6 illustrates the patient‐level classification accuracy of different models, along with the average deviation between the predicted probability values and the true values. The comparison reveals that among the lightweight models, all except the proposed CopFSNet perform significantly worse than the standard models, with a larger accuracy gap between the internal and external test sets. In contrast, our lightweight CopFSNet model achieves the highest accuracy of 91.43% and 85.95% on the internal and external test sets, respectively, the average prediction deviations of 0.1567 and 0.1965, and the smallest accuracy gap of 5.48% between the internal and external test sets. These results highlight the strong classification performance of our model at the patient level and underscore its promising potential for reliable clinical application.

### 3.5 Additional Statistical Evaluation and Supplementary Results

We incorporate 95% confidence intervals and conducted statistical significance tests for all baseline comparisons in the image‐level classification task. Furthermore, calibration curves, Brier scores, and decision threshold sensitivity analyses are added to provide a more comprehensive and reliable evaluation of model performance. All corresponding results are included in the Supporting Materials.

In addition, we perform a comprehensive ablation study to quantify the individual contributions of the FDA, FEP, and DGA modules, as well as the pruning operation and the feature sharing mechanism, for both the segmentation and classification tasks. The full ablation results and supporting materials are also provided in the Supporting Materials.

### 3.6 Model Efficiency

**Figure 7:**
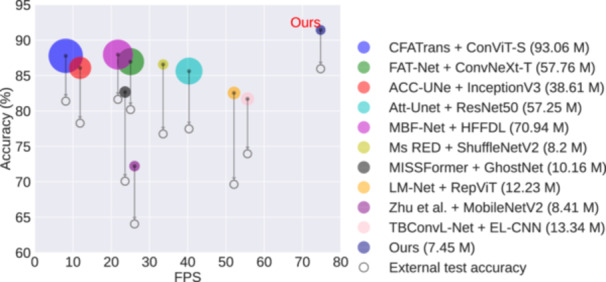
Visual comparison of classification performance and inference speed at the patient level for different model combinations. The diameter of the circle is proportional to the total number of parameters of the corresponding combination in the legend. FPS, frames per second.

The proposed CopFSNet model not only achieves high segmentation and classification performance but also exhibits a smaller model size and faster inference speed. For the segmentation task, CopFSNet demonstrates an impressive inference speed of 115.83 FPS, surpassing other lightweight models such as FF‐UNet and LM‐Net (Table 2). In the classification task, CopFSNet operates with just 3.42 M parameters and a computational cost of 0.67 GFLOPs, achieving an inference speed of 85.35 FPS (Table 4). Figure 7 illustrates the scale of the combined segmentation and classification models, along with a comparison of classification accuracy at the patient level. It is evident that, except for our proposed model, the performance of other models tends to decline as FPS increases. However, our proposed model overcomes this limitation, achieving the highest classification performance and fastest inference speed while maintaining a compact size.

## 4 Discussion

In this study, we developed and validated CopFSNet, a deep learning model designed to automatically segment ultrasound images of hepatic echinococcosis and classify them into AE and CE categories. Validation results on both internal and external test datasets demonstrate that CopFSNet achieves state‐of‐the‐art performance in segmentation and classification at both the image and patient levels. Notably, its robust performance extends to diverse settings, including high‐burden regions for hepatic echinococcosis and tertiary hospitals. Furthermore, the model's compact size and high inference speed make it an optimal choice for deployment on clinical diagnostic devices, supporting efficient and reliable diagnosis.

CopFSNet's accurate and robust performance, combined with its lightweight model architecture and high inference speed, makes it highly suitable for deployment in clinical settings. Specifically, the model is well‐suited for disease screening in frontline endemic regions of hepatic echinococcosis, where rapid and reliable diagnostic tools are essential. Furthermore, its robustness ensures it can serve as a dependable clinical decision‐support tool in tertiary hospitals. Previous research has primarily focused on developing CT‐based AI systems for hepatic echinococcosis screening [19]. However, CT systems not only expose patients to additional radiation during the screening process but are also challenging to implement in underdeveloped regions where endemic areas of echinococcosis are often located [54]. In contrast, low‐cost, portable ultrasound devices offer a more practical and accessible solution for screening at the community level, making them an ideal choice for resource‐limited settings despite the examination and diagnosis heavily depends on the ultrasound radiologist's skill [55, 56]. Therefore, the development of an accurate and lightweight deep learning model for hepatic echinococcosis segmentation and classification on ultrasound images could significantly enhance screening efforts in rural areas, improving access to reliable diagnostic tools where they are needed most.

In the current study, CopFSNet demonstrates exceptional performance in both segmentation and classification tasks, achieving the highest Dice value of 80.67% and surpassing both standard [31, 32, 33, 34, 35, 36, 37] and lightweight [38, 39, 40, 41, 42, 43] models across all evaluation metrics. This improvement stems from the integration of the FDA and DGA modules, which enhance feature extraction at different spatial scales. The FDA module focuses on detailed lesion‐specific features, while the DGA module captures broader global context, and their combined outputs ensure precise and consistent lesion delineation. Additionally, the FEP module selectively emphasizes relevant self‐attention computations, further optimizing feature representations. With systematic pruning, the model achieves a peak Dice value of 80.71%, and its strong generalization ability is reflected by a Dice score of 78.12% on the external test set. For classification tasks, CopFSNet leverages segmentation‐derived features to enhance lesion recognition and subtype differentiation, achieving over 7% higher classification accuracy compared to ResNet50 [44] and outperforming other models in AUC. It achieves the highest patient‐level accuracy, with 91.43% on the internal test set and 85.95% on the external test set, while maintaining minimal prediction deviations. These results highlight CopFSNet's robustness, making it a valuable diagnostic tool for hepatic echinococcosis. Its strong generalization to external datasets indicates potential for deployment in diverse healthcare settings, from rural screening in endemic regions to advanced diagnosis in tertiary hospitals. As the spread of hepatic echinococcosis increases with expanding transportation networks, reliable tools like CopFSNet are essential for early detection and global disease management.

Methodologically, most prior multi‐stage or multi‐resolution approaches treat segmentation [57, 58, 59] and classification [60, 61] as separate sequential processes, often relying on cascaded models or multi‐scale refinement stages that increase computational complexity. In contrast, our framework employs a unified architecture in which segmentation features are directly shared to assist the classification task. Rather than constructing multiple‐resolution pathways or multi‐stage refinement, our method leverages the structural information captured by the segmentation encoder to enhance lesion discrimination in a single streamlined pipeline.

Practically, existing multi‐stage/multi‐resolution approaches typically require heavier computation and longer inference times [62, 63], which limit deployment on portable ultrasound devices and in resource‐limited settings. Our model is lightweight by design and achieves real‐time inference while maintaining high segmentation and classification performance. This practical advantage enables large‐scale screening and supports clinical use in remote pastoral regions where hepatic echinococcosis is endemic.

A common concern with using ultrasound as the primary technology for hepatic echinococcosis screening is the need for experienced ultrasound radiologists, while there is often a shortage of skilled healthcare workers in endemic regions [64, 65]. As a result, many studies have focused on enhancing the performance of detecting and subtyping hepatic echinococcosis from selected frames of ultrasonography [66, 67]. However, ultrasound radiologists still need to manually locate and identify lesions. To tackle this challenge, our proposed CopFSNet achieves near‐real‐time segmentation and subtyping with a frame rate of 74.76 FPS for the joint task. This capability to capture potential hepatic echinococcosis lesions in real time is especially important, as lesions may appear fleetingly on the ultrasound device, requiring rapid detection. This capability to capture potential hepatic echinococcosis lesions in real time is especially important, as lesions may appear fleetingly on the ultrasound device, requiring rapid detection. Achieving high efficiency, CopFSNet uses several architectural innovations. Its lightweight design optimizes feature maps at different downsampling levels: 16× downsampled features are processed with a FDA module, and 4× downsampled features with a DGA module, balancing performance and model size for fast inference. Additionally, the FEP module accelerates inference by pruning redundant self‐attention calculations, reducing computational load, and refining lesion delineation. This combination ensures high frame rates and superior segmentation performance.

Our study has several limitations. Firstly, while a substantial dataset of 20,112 internal images and 3340 external images was used, the data originated from two centers in Sichuan Province, China. This geographical specificity may limit the generalizability of the model to populations with different epidemiological characteristics or variations in ultrasound imaging protocols and equipment used in other regions. Although the external validation using data from West China Hospital provided some evidence of generalizability, further validation on more diverse datasets from multiple centers globally is warranted. Secondly, all images included in the study contained only one type of lesion (CE or AE) and a single region of interest. This simplified the segmentation and classification tasks compared to real‐world clinical scenarios where patients might present with multiple lesions or co‐infections. Future research should investigate the model's performance in more complex cases involving multiple lesions or the co‐occurrence of CE and AE. Thirdly, although efforts were made to standardize image acquisition and annotation, inherent variability in ultrasound image quality due to operator experience, patient body habitus, and equipment settings could have introduced noise into the data. Although the model demonstrated robustness on the external test set, further investigation is required to evaluate the influence of these factors on its performance. Finally, while the model achieved high accuracy in both segmentation and classification tasks, its clinical utility needs to be validated in prospective clinical studies to assess its impact on diagnostic accuracy, workflow efficiency, and patient outcomes.

## 5 Conclusion

In this study, we have developed CopFSNet, a novel deep learning model for the dual tasks of segmentation and classification of hepatic echinococcosis in ultrasound imaging. This model demonstrates high accuracy and efficiency in both lesion segmentation and disease subtyping (CE and AE), outperforming several standard and lightweight deep learning models. Its compact architecture, characterized by a low number of parameters and fast inference speed, makes it highly suitable for deployment on clinical ultrasound devices, particularly in resource‐limited settings, as well as in tertiary hospitals in developed regions. By providing accurate lesion segmentation along with image‐level and patient‐level classification, our model has the potential to significantly assist ultrasound radiologists in interpreting ultrasound images, streamline diagnostic workflows, and improve the accuracy of hepatic echinococcosis diagnosis. This work addresses the critical need for automated diagnostic tools in regions with limited access to experienced ultrasound radiologists, while also offering valuable support in more advanced healthcare settings. Furthermore, the methodology employed in this study, such as the integration of frequency‐domain information and the use of segmentation feature maps to enhance classification, can be adapted and applied to other medical image analysis tasks. Future work will focus on prospective clinical validation in larger, more diverse populations and exploring the integration of this model into existing clinical workflows to fully realize its potential impact on patient care, ultimately improving the diagnosis of hepatic echinococcosis in both endemic and developed regions.

## Author Contributions

**Zhu He:** methodology, investigation, writing – original draft, visualization (equal). **Jiajun Qiu:** conceptualization, data curation, resources, project administration, supervision (equal). **Chenlin Du:** writing – methodology, review and editing, validation, investigation (equal). **Jin Yin:** investigation, resources (equal). **Xuhui Zhang:** investigation, resources (equal). **Yelei Ren:** investigation, resources (equal). **Yifei Wang:** investigation, resources (equal). **Lamu Suolang:** investigation, resources (equal). **Chunyang Li:** investigation, resources, funding acquisition (equal). **Zongjiu Zhang:** project administration, supervision, validation (equal). **Diming Cai:** conceptualization, supervision, funding acquisition (equal). **Qicheng Lao:** investigation, writing review and editing, project administration, supervision (equal).

## Ethics Statement

The study protocol was approved by the Ethics Committee of West China Hospital, Sichuan University (Approval 2019482).

## Consent

Due to the retrospective nature of the study, the requirement for written informed consent was waived.

## Conflicts of Interest

Professor Zongjiu Zhang is a member of the *Health Care Science* Editorial Board. To minimize bias, he was excluded from all editorial decision‐making related to the acceptance of this article for publication. The remaining authors declare no conflicts of interest.

## Supporting information

Supporting File

## Data Availability

The data are not publicly available due to privacy or ethical restrictions.
