# Supplementary material for: Development and External Validation of an Efficient Deep Learning Model for Lesion Segmentation and Subtyping of Hepatic Echinococcosis in Ultrasound Imaging
Source: Health Care Sci. 2026 May 19:10.1002/hcs2.70077. Online ahead of print. doi: 10.1002/hcs2.70077 (PMC13399102; doi:10.1002/hcs2.70077)
Supplement: Supplementary file 1 — Supporting File [file HCS2-9999-0-s001.docx]

**Supplementary Materials**

**1 Supplementary Methods**

The proposed CopFSNet model consists of three main parts, namely, a frequency-domain attention (FDA) module for modeling the context dependencies in frequency- and spatial-domain, followed by a frequency excitation and pruning (FEP) module for self-attention enhancement and pruning, and finally a deformable global attention (DGA) module for fast feature downsampling. By leveraging the coupled context dependencies in frequency- and spatial-domain and constructing a feature pyramid with different levels, our model is able to efficiently extract not only local features with important frequency domain information but also capture important global context information, facilitating the segmentation and classification of hepatic echinococcosis in ultrasound images.

**1.1 Frequency domain attention module**

The Discrete Fourier Transform (DFT) is extensively employed in signal processing for signal acquisition and measurement [1]. Given an image $X$, its width and height are $M$ and $N$, respectively, and the pixels are represented by $x_{m,n}$ ($1\leq m\leq M,1\leq n\leq N$). The process of 2D DFT for this image is as follows:

where $j$ is the imaginary unit. For each pixel $x_{m,n}$, Eq. (1) performs discrete Fourier transforms in the dimensions of height and width respectively. DFT satisfies the property of conjugation symmetry, that is, $x'_{M-u,N-v}=x'_{u,v}$. Combined with the periodicity properties of $e^{-j2\pi\left(\frac{um}{M}+\frac{vn}{N}\right)}$, the computational complexity of transforming an image from the spatial domain to the frequency domain can be reduced to , which is called the fast Fourier transform (FFT) [2]. Furthermore, the DFT is reversible, for the values $x'_{u,v}$ in the frequency domain, according to the following formula:

the pixel value $x_{m,n}$ in the original image can be obtained.

In our proposed FDA module, features with 16$\times$ downsampling size are first transformed to tokens by patch embedding, thus realizing feature fusion on the channel dimension. Let $C$ be the number of channels of the feature maps. Each feature map $f_{t}$ ($1\leq t\leq C$) is transformed to the frequency domain by FFT, i.e., . Then multiply $F_{t}$ element by element with a learnable filter $w_{t}^{f}$:

$$F'_{t}=F_{t}\odot w_{t}^{f},$$

where , and $M'$, $N'$ represent the width and height of the feature maps, respectively. The parameters in filter $w_{t}^{f}$ are updated by a back-propagation algorithm. After that, it is transferred to the space domain through the inverse Fourier transform, i.e., , so as to realize the update of the feature map $f_{t}$. In this way, different frequency domain components of features can be captured by the above transformation through the learnable filter $w_{t}^{f}$. The output is then passed through a feedforward (FFD) network, which realizes the fusion of channel information after the frequency domain transformation, thereby enhancing the localization of the frequency domain feature extraction. The whole process can be expressed using the following formula:

where represents the transformation of the input by the linear layers and ReLU layer in the FFD network.

Finally, the feature maps $\tilde{f}$ after the above transformation enter into the multi-head self-attention (MHSA) module [3]. The process of self-attention computation can be represented as follows:

$$Q=\tilde{f}\cdot W_{q}, K=\tilde{f}\cdot W_{k}, V=\tilde{f}\cdot W_{v},$$

$$SA\left(Q,K,V\right)=\text{Softmax}(\frac{QK^{\top}}{\sqrt{d_{k}}})V=\text{Softmax}\left(\frac{\left(\tilde{f}\cdot W_{q}\right)\cdot\left(\tilde{f}\cdot W_{k}\right)^{\top}}{\sqrt{d_{k}}}\right)\cdot\tilde{f}\cdot W_{v}.$$

In Eq. (5), , $h$ is the number of heads, and we set $d_{k}=3\cdot C/h$. The frequency domain transformed feature map $\tilde{f}$ is multiplied with the learnable transformation matrix $W_{q}$, $W_{k}$, $W_{v}$, thus mapping to the space of query ($Q$), key ($K$), and value ($V$), respectively. After that, Eq. (6) first calculates the correlation between $Q$ and $K$, and then multiplies it with $V$ to get the result of self-attention.

According to Eq. (2) and(3), we can obtain the following equation:

where $W^{f}=\left(w^{f}_{1},w^{f}_{2},\cdots,w^{f}_{C}\right)$, $\tilde{F}=\left(F_{1},F_{2},\cdots,F_{C}\right)$. Therefore, Eq. (6) can be calculated as follows:

$$\begin{matrix} \text{SA}\left(Q,K,V\right)=\text{Softmax}\left(\frac{\left(\Phi_{W^{f}}\left(\tilde{F}\right)\cdot W_{q}\right)\cdot\left(W_{k}^{\top}\cdot\left(\Phi_{W^{f}}\left(\tilde{F}\right)\right)^{\top}\right)}{\sqrt{d_{k}}}\right) \\ =\text{Softmax}\left(\frac{\Phi_{W^{f}}\left(\tilde{F}\right)\cdot\hat{W}_{q,k}\cdot\left(\Phi_{W^{f}}\left(\tilde{F}\right)\right)^{\top}}{\sqrt{d_{k}}}\right)\cdot\Phi_{W^{f}}\left(\tilde{F}\right)\cdot W_{v}, \end{matrix}$$

where $\hat{W}_{q,k}=W_{q}\cdot W_{k}^{\top}$. In Eq. (8), the scaled dot-product of $\Phi_{W^{f}}\left(\tilde{F}\right)$ with itself makes the frequency domain feature $W_{f}$ have global attention over all features, which we call frequency-domain attention. The channel locality of and the global correlation of $SA\left(\cdot\right)$ complement each other, therefore, the combination of frequency domain transformation and self-attention mechanism can perform better feature extraction. The MHSA calculates self-attention separately using multiple subgroups according to the following equation:

$$\text{MHSA}=\text{Concat}\left[\text{SA}_{1},\text{SA}_{2},\cdots,\text{SA}_{h}\right]W^{0},$$

where . This provides more different patterns of frequency-domain attention. After that the output is again passed through the FFD network for information fusion in the channel dimension and the final output of the FDA module is obtained. To enhance the frequency domain attention modeling capability, we stack the FDA module twice the proposed model.

In the above MHSA computation process, the linear mapping computational complexity is , the complexity of the self-attention computation process is and the computational complexity of the linear mapping of the multi-head outputs is . The total computational complexity is . In the process of frequency domain feature extraction, the computational complexity of FFT and iFFT is , and the computational complexity of multiplying with the weights $W$ is . The total computational complexity is . We use hyper-parameters $d_{k}$ of 64 and $h$ of 8, and $n=14$, thus the computational complexity of the frequency-domain operation is significantly lower than that of the self-attention operation.

**1.2 Frequency excitation and pruning module**

Frequency domain feature modeling can replace the self-attention mechanism for image recognition, and the combination of the two can obtain better performance than a single structure [4]. However, while $\Phi_{W^{f}}\left(\cdot\right)$ and $\hat{W}_{q,k}$ in Eq. (8) strengthen the nonlinear expression of the FDA module, they also weaken the correlation between the frequency domain feature extraction and the self-attention modeling, and increase the learning difficulty of the parameter $W^{f}$, which reduces the convergence speed of the model. Therefore, we propose a frequency excitation and pruning (FEP) module, which is used to enhance the coupling between frequency domain modeling and self-attention calculations. The FEP module takes the weight parameter $W^{f}$ as input, and the computational process can be represented as follows:

$$\text{Gate}\left(x\right)=\left\{\begin{matrix} x,\text{ }x>a, \\ 0,\text{ }x\leq a, \end{matrix}\right.$$

where is used to represent the combination of layer normalization, linear layer, ReLU function, and Sigmoid function in the FEP module. We multiply the output of the by two before it is gated so that the mean of the outputs of the Sigmoid activation function becomes one. Thus, the influence of FEP on self-attention modeling under parameter initialization is reduced. The $Gate(\cdot)$ function masks the output of the FEP module by setting the hyperparameter $\alpha$. , denotes the weight matrix after pruning mask, which indicates the importance of different self-attention relationship modeling from the perspective of the frequency domain.

It should be noted that the FDA module uses feature maps with 16$\times$ downsampling as input, while the convolutional layer itself extracts many redundant feature maps [5]. In addition, for ultrasound images of hepatic echinococcosis, it is also redundant to model parts outside the observation area and not related to the focal area. Therefore, we propose to use frequency-domain attention pruning based on the frequency-domain parameter $M^{f}$. Specifically, the redundancy of attention modeling without obvious frequency domain patterns is filtered out through gating in Eq. (10). Following the common practice of pruning the $Q$-space in the MHSA calculations, we block the redundant attention and excite the critical attention by multiplying the gating parameter $M^{f}$ in the $Q$-space of the MHSA, calculated as follows:

$$M^{f}=\left(m_{1},m_{2},...,m_{t}\right),$$

$$W_{q}=\left(w_{1}^{q},w_{2}^{q},...,w_{t}^{q}\right),$$

$$W_{q}^{'}=M^{f}\odot W_{q}=\text{Gate}\left(m_{i}\cdot w_{i}^{q}\right),$$

where $i=1,2,...,t$. When $w_{i}^{q}\geq\alpha$, the corresponding $m_{i}$ is excited; otherwise, the corresponding $m_{i}$ is pruned. We construct a one-way strong mapping relation from the frequency-domain parameter $M^{f}$ to the $Q$-space in the MHSA by means of Eq. (10) and Eq. (14). The parameter $M^{f}$ is only used as an input to the FEP module, and the gradient is not propagated to this parameter during the back propagation process of the FEP module, thus avoiding a negative impact on the FDA module on the backbone. As a result, the strong coupling between contextual dependencies in frequency- and spatial-domain can be constructed in the FEP module.

**1.3 Deformable global attention module**

Multi-level feature extraction is widely used to enhance the perception of lesion regions with different sizes [6][7]. Utilizing the FDA with the FEP module for different levels of features will undoubtedly increase the size of the model significantly, thus we propose to use the deformable global attention (DGA) module with fast feature downsampling. The DGA module uses the features from the 4$\times$ downsampling as input and uses patch projection to further 4$\times$ downsampling, thus allowing the output of the DGA module to be consistent in size with the output of the FDA module. In the DGA module, MHSA and deformable attention are used alternately in order to better model the attention between the feature map tokens.

The biggest difference between deformable attention and MHSA is that the former has additional learnable locations of the key-value pairs. The corresponding query, key, and value calculation process in the deformable attention are as follows:

$$q=W_{q}^{d}\cdot x, \tilde{k}=W_{k}^{d}\cdot\tilde{x}, \tilde{v}=W_{v}^{d}\cdot\tilde{x},$$

where $F_{o}$ is a subnetwork of learning offset. Eq. (16) follows the same process as the standard MHSA calculation. A new feature $\tilde{x}$ is obtained by bilinear interpolation on the feature at position $p+F_{o}\left(q\right)$, thus replacing the feature $x$ at the original position $p$. is a bilinear interpolation sampling function with differentiability, which is calculated as follows:

where $\left(r_{i},r_{j}\right)$ indexes all the locations on and $z\left(x,y\right)=\max\left(0,1-\left|a-b\right|\right)$. Since $g$ is non-zero only at the four integration points closest to$\left(p_{x},p_{y}\right)$, it reduces Eq. (17) to a weighted average of the four positions. We then perform self-attention for $q$, $\tilde{k}$, and $\tilde{v}$ with relative positional offsets $R$ as follows:

Similar to Eq. (9), we can obtain the formula for the multi-head self-attention as follows:

$$\text{DA-MHSA}=\text{Concat}\left[\text{DA-SA}_{1},\cdots,\text{DA-SA}_{h^{d}}\right]W_{o}^{d},$$

where . The DGA module is used to model globally important regions and to focus on significant features that lack valid frequency domain information.

It is worth noting that we use patch embedding to process the input in the FDA module, while the patch projection operation is used in the DGA module. The similarity between the two is that both expand features in the channel dimension. The difference is that the former directly reconstructs the features without changing the input size, while the latter is essentially equivalent to a convolution operation that 4$\times$ downsampling the features. In this way, feature pyramids with different hierarchies can be constructed, avoiding repeated self-attention modeling of the same features.

**1.4 Loss function in model training**

We use binary cross entropy loss ($L_{CE}$) to calculate the pixel level segmentation loss value and add it with Dice loss ($L_{Dice}$) in order to get better segmentation results. The total segmentation loss value $L_{Seg}$ is calculated as follows:

$$L_{CE}=-\frac{1}{n}\sum_{i=1}^{n} \left(y_{i}\text{ln}\left(p_{i}\right)+\left(1-y_{i}\right)\text{ln}\left(1-p_{i}\right)\right),$$

$$L_{Dice}=1-\frac{2\times\sum_{i=1}^{n} \left(p_{i}\cdot y_{i}\right)}{\sum_{i=1}^{n} p_{i}+\sum_{i=1}^{n} y_{i}},$$

$$L_{Seg}=L_{CE}+L_{Dice},$$

where $n$ denotes the number of pixels, $y_{i}$ denotes the label of the $i\text{-th}$ pixel, and $p_{i}$ denotes the predicted probability value of the $i\text{-th}$pixel belongs to the lesion regions.

Figure S1 shows the detailed structure of solving the classification task using the partition-trained CopFSNet model. After freezing the parameters of the segmentation encoder of the CopFSNet, we take out the middle feature maps as the partial feature maps of the classification part Similar to Eq. (20), the classification part is trained by calculating the following classification cross-entropy loss ($L_{Cls}$) using the classification labels. The specific calculation procedure for $L_{Cls}$ is as follows:

$$L_{Cls}=-\frac{1}{N}\sum_{j=1}^{N} \left(y'_{j}\text{ln}\left(p'_{j}\right)+\left(1-y'_{j}\right)\text{ln}\left(1-p'_{j}\right)\right),$$

where $N$ denotes the number of images, $y'_{j}$ denotes the label of the $j\text{-th}$image, and $p'_{j}$ denotes the predicted probability of the $j\text{-th}$ image belongs to the AE category. This step-by-step approach to solving dual tasks can reduce the complexity of the problem without considering the problem of gradient normalization [8].


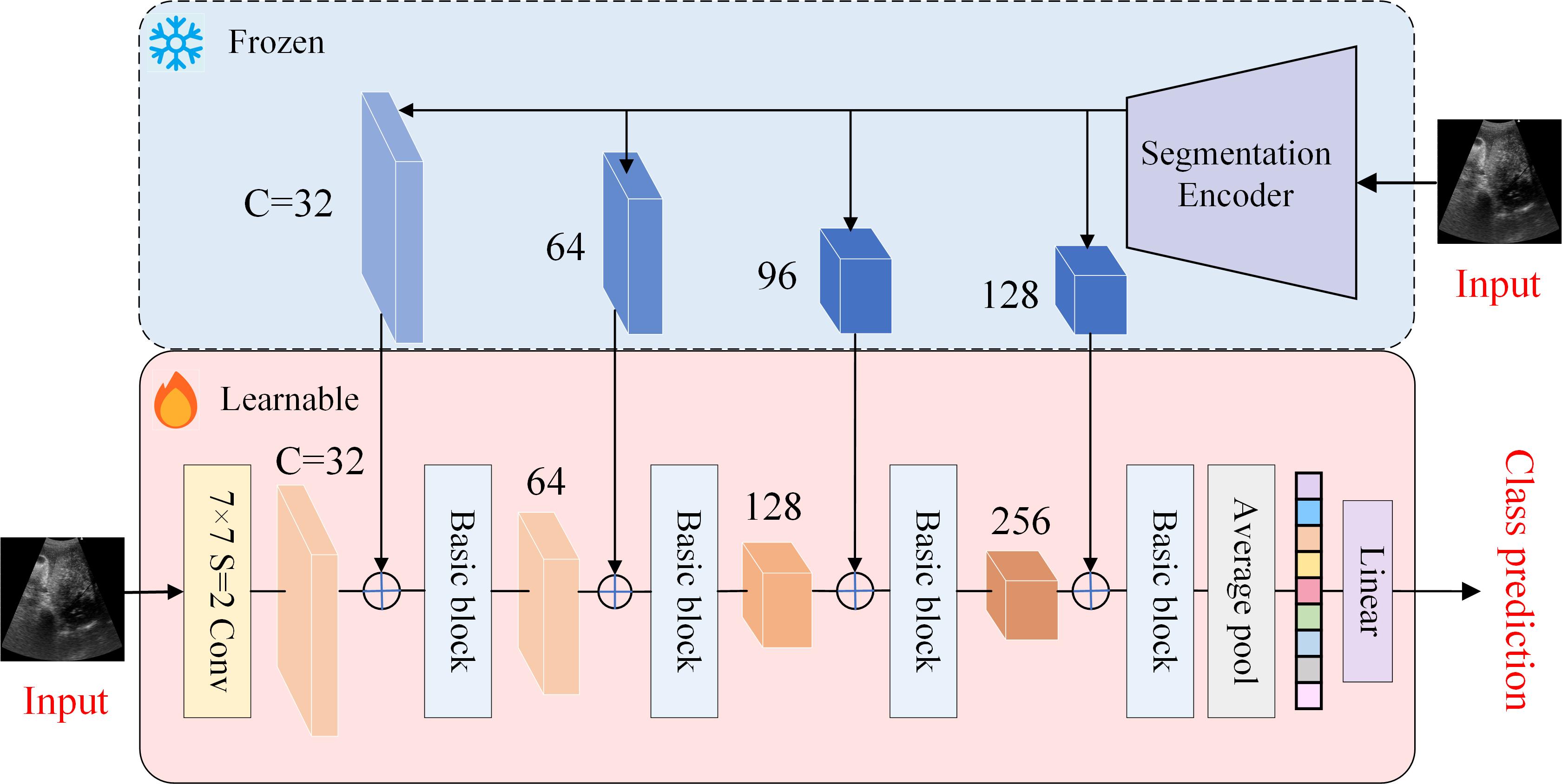


Fig. S1 The structure of the CopFSNet model for the classification task. The model parameters of the segmentation part are frozen, and the model parameters of the classification part are activated.

**1.5 Ultrasound Imaging and Post-Processing**

**Ultrasound devices and probes:** Ultrasound images were acquired using the Biosound MyLab™ X7 system with an abdominal convex probe AC2541 (8–1 MHz), the Philips CX50 portable system with an abdominal convex probe C5-1 (5–1 MHz), and the Mindray Z60 portable color Doppler system with an abdominal convex probe C6-2P (6–2 MHz).

**Acquisition protocols:** Images were obtained following standard abdominal scanning procedures.

**Image post-processing:** No additional filtering or denoising was applied beyond standard device post-processing. To reduce overfitting during training, we employed online data augmentation, including random scaling of images within the range [0.8, 1.1] and horizontal flipping with a 50% probability. The images were then resized to 256×256 pixels and center-cropped to 224×224 pixels as model input, removing redundant regions not relevant to the diagnosis of hepatic echinococcosis. No processing was applied to images in the validation and test sets, ensuring that the model’s segmentation results remain accurate and reliable.

**1.6 Training and Optimization Details**

For optimization, we employ the AdamW optimizer together with a cosine annealing learning rate schedule and an early stopping strategy. In the segmentation task, the maximum learning rate is set to 1e-4, with a maximum number of 200 training epochs, the warm up epoch is 10, and the batch size is 32. For the classification task, a warm-up period of 10 epochs, and a batch size of 32. For the classification task, the maximum learning rate is set to 1e-3, with 100 training epochs, a warm-up period of 5 epochs, and a batch size of 64. Since the classification model utilizes feature maps generated by the segmentation encoder, fewer training iterations are required. Early stopping is applied when the validation loss fails to decrease for eight consecutive epochs. All experiments are conducted on a Windows-based server equipped with two NVIDIA Tesla V100S GPUs, each with 32 GB of memory.

**2 Supplementary Results**

**2.1 Statistics on Lesion Distribution**

Figure S2(A) shows the distribution of lesion sizes in AE and CE images. Combined with Figure S2(B), AE images have a noticeably higher proportion of small lesions occupying less than 5% of the image area compared to CE. For medium-sized lesions (5–15% of the image area) and large lesions (over 15% of the image area), the distributions of AE and CE are largely similar.

Figure S2(C) presents the two-dimensional kernel density estimation maps of the normalized lesion centroid positions for CE and AE groups. The heat intensity reflects the spatial concentration of lesion centroids within the imaging plane, where brighter regions represent higher density. Coordinates are normalized to the image size (0–1 range). The normalized centroid heatmaps of AE and CE lesions show broadly similar spatial distributions, with both categories concentrated near the central region of the image. AE lesions exhibit a slightly more compact and symmetric distribution, whereas CE lesions display a marginally wider spread along the horizontal axis, indicating greater variability in lateral positioning. Overall, both types predominantly cluster around the mid‐field region, with no extreme positional bias toward image boundaries.

Figure S2(D) shows the distribution of shape features in lesion regions of AE and CE images. It can be seen that both types exhibit a general negative correlation between eccentricity and circularity, with most shapes roughly close to circular or elliptical. CE lesions tend to be more regular and concentrated, whereas AE lesions are more diverse, including a higher proportion of elongated and irregular shapes. However, due to substantial overlap between the two types in most regions, eccentricity and circularity are unlikely to serve as effective features for distinguishing AE from CE.


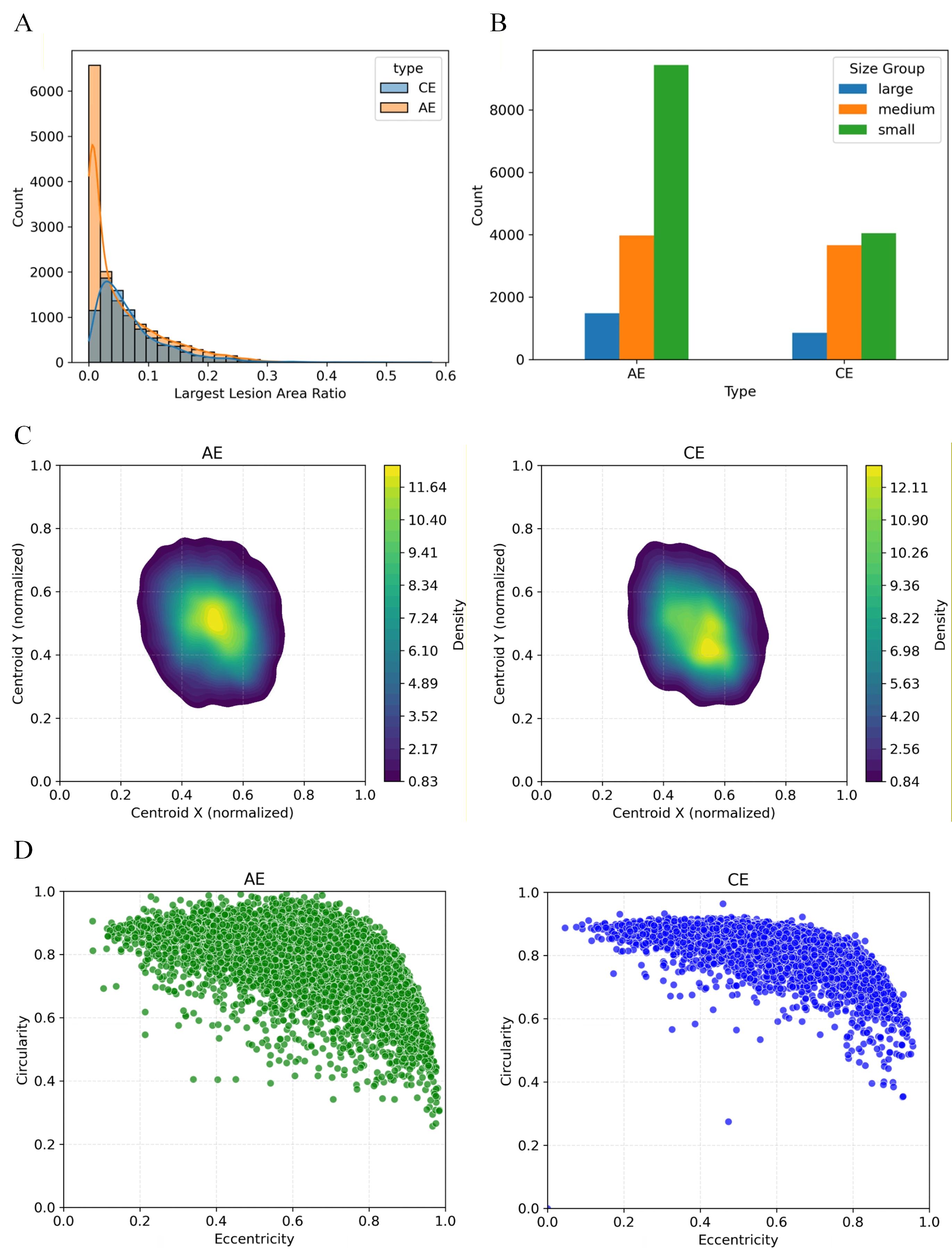


**Fig. S2** Stratified statistics of AE and CE lesions. (A) Distribution of lesion areas as a proportion of the total image size. (B) Number of lesions grouped by size, classified as small, medium, or large based on thresholds of 0.05 and 0.15 of the image area. (C) Normalized centroid location distributions for CE and AE lesions. (D) Distribution of lesion shape characteristics in CE and AE cases

**2.2 Confidence Intervals and Statistical Tests**

Table S1 reports the classification accuracy, Brier scores, and corresponding 95% confidence intervals for different models. The results demonstrate that the proposed CopFSNet achieves the highest accuracy and the lowest Brier score on both the internal and external test sets, indicating superior discriminative performance and better-calibrated probability predictions.

**Table S1** Classification accuracy and 95% confidence intervals (95% CI) of different models on the image classification task

| model | Accuracy (95% CI) | Brier score (95% CI) |
| --- | --- | --- |
| Internal test |  |  |
| MobileNetV2 | 0.6738 (0.6544-0.6937) | 0.2177 (0.2109-0.2236) |
| GhostNet | 0.7966 (0.7787-0.8135) | 0.1435 (0.1334-0.1538) |
| ShuffleNetV2 | 0.8359 (0.8195-0.8528) | 0.1176 (0.1067-0.1287) |
| RepViT | 0.7882 (0.7703-0.8066) | 0.1471 (0.1371-0.1566) |
| EL-CNN | 0.7445 (0.7246-0.7628) | 0.1788 (0.1689-0.1880) |
| Ours | 0.9010 (0.8876-0.9140) | 0.0754 (0.0672-0.0846) |
| External test |  |  |
|  |  |  |
| MobileNetV2 | 0.5994 (0.5823-0.6159) | 0.2382 (0.2336-0.2422) |
| GhostNet | 0.6581 (0.6413-0.6749) | 0.2384 (0.2279-0.2488) |
| ShuffleNetV2 | 0.7132 (0.6988-0.7288) | 0.2154 (0.2044-0.2262) |
| RepViT | 0.6659 (0.6503-0.6817) | 0.2315 (0.2215-0.2413) |
| EL-CNN | 0.6722 (0.6566-0.6889) | 0.2155 (0.2079-0.2229) |
| Ours | 0.8096 (0.7964-0.8234) | 0.1538 (0.1439-0.1635) |

Figure S3 presents the calibration plots for all models on the image classification task. It can be observed that CopFSNet exhibits the closest alignment between predicted confidence and actual outcomes across both test sets, suggesting more reliable confidence score distributions when distinguishing AE from CE.

Furthermore, Figure S4 provides the decision-threshold sensitivity analyses of different models. As the decision threshold varies, CopFSNet consistently maintains higher sensitivity and specificity compared with other baselines, while achieving a more favorable balance between the two metrics on both the internal and external test sets.

Together, these results indicate that CopFSNet delivers robust, accurate, and well-calibrated classification performance for distinguishing AE and CE images.


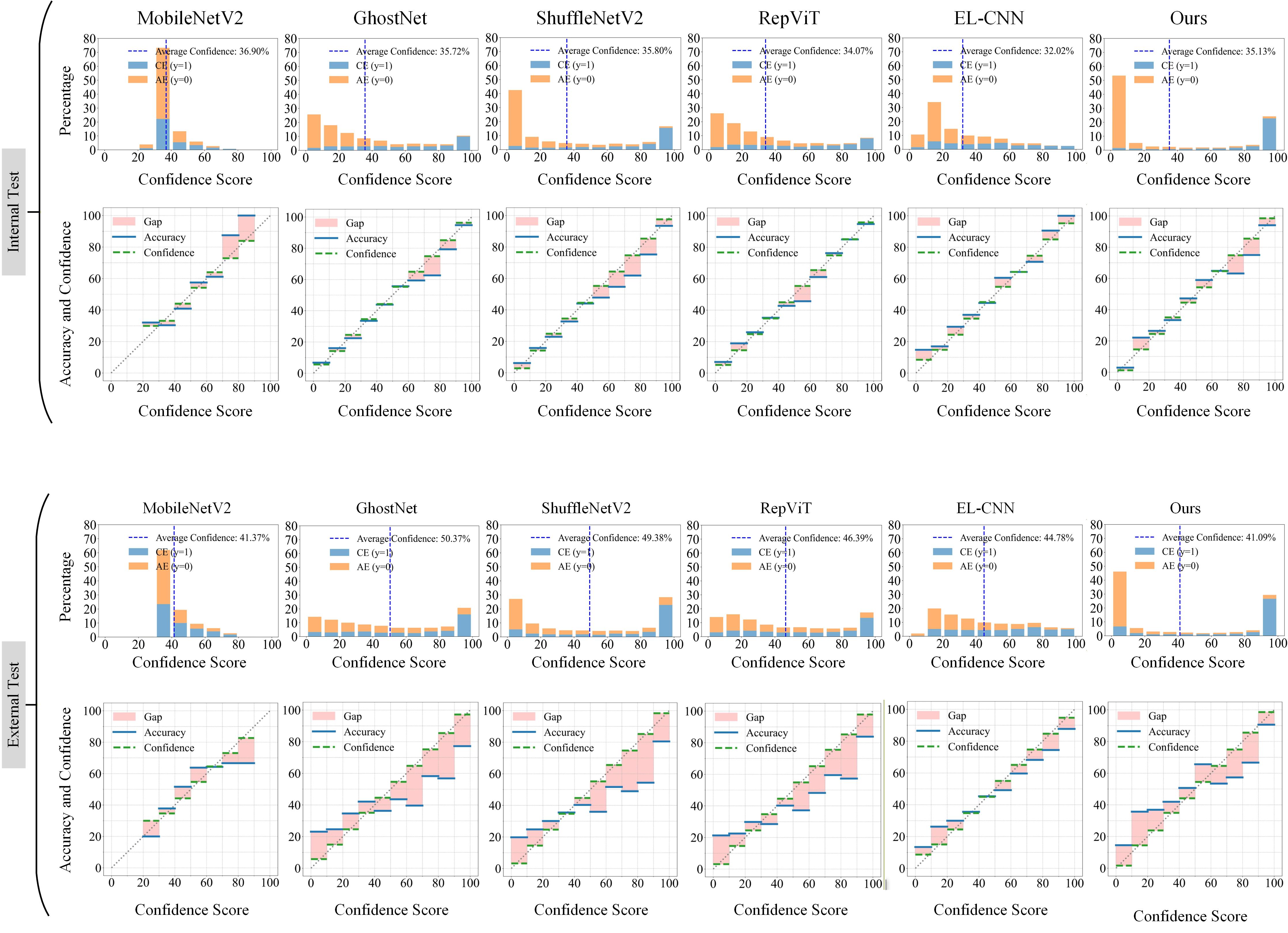


**Fig. S3** Calibration plots of different models on the image classification task. In both the internal and external test sets, we present two rows of tables. The first row contains bar charts that display the proportion of samples at different confidence scores, along with the average confidence (blue vertical line). The second row shows the accuracy and average confidence of samples within each of the ten bins. Ideally, the gap between accuracy and average confidence should be zero


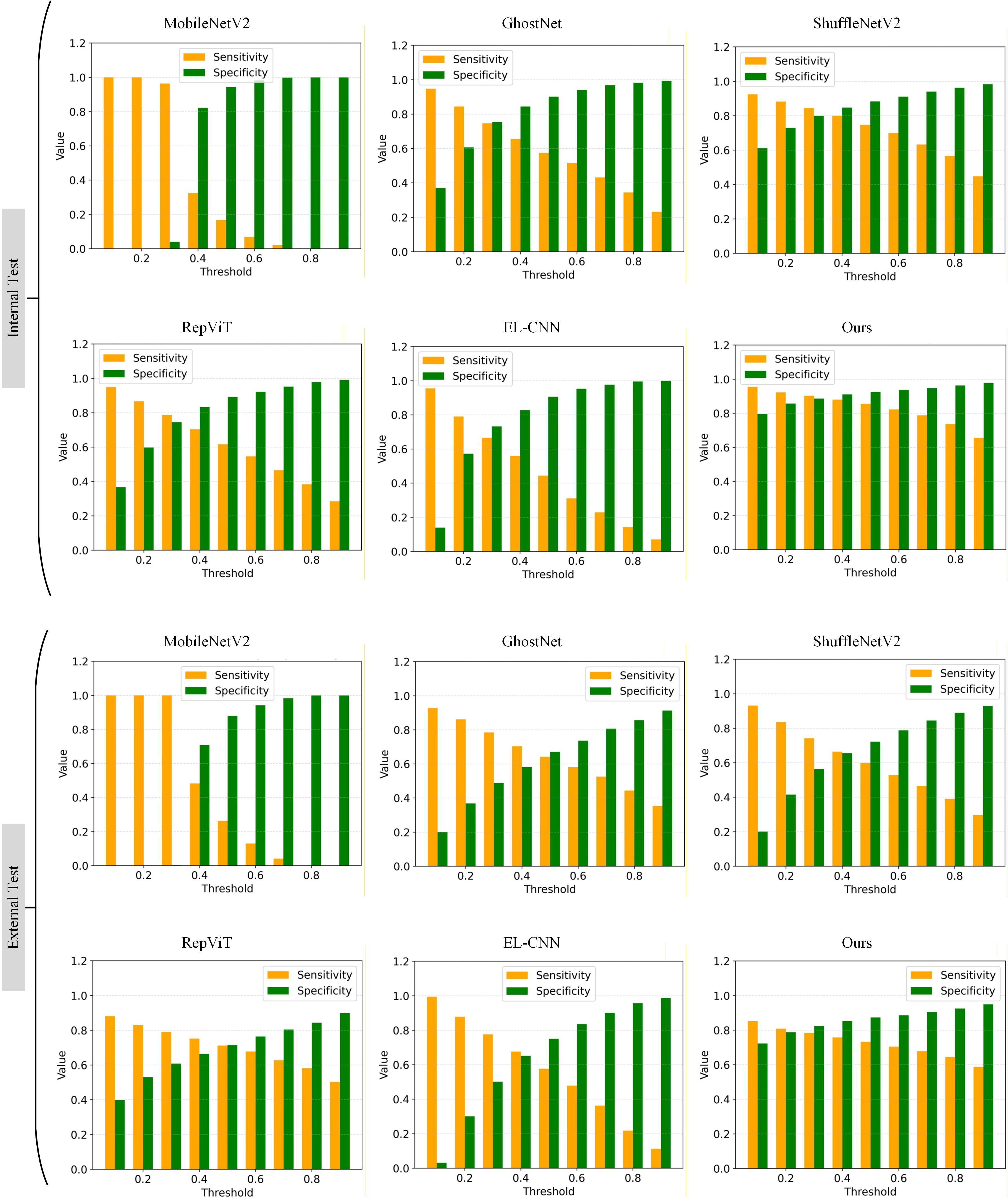


**Fig. S4** Decision-threshold sensitivity analyses of different models on the image classification task

**2.3 Ablation study**

We conducted a comprehensive ablation study to quantify the individual contributions of the FDA, FEP, and DGA modules, as well as the pruning operation and feature sharing mechanism. The results are presented in Tables S2, S3, and S4.

As shown in Table S2, for the segmentation task, incorporating the three modules together with the pruning operation led to consistent performance gains, improving the Dice score from 77.02% and 74.13% to 80.12% and 78.12% on the internal and external test sets, respectively. Table S3 reports the ablation results for the image-level classification task. Since the FDA, DGA, and FEP modules, as well as pruning, influence classification through the shared feature maps from the segmentation encoder, a similar trend was observed, with classification accuracy increasing from 85.75% and 74.28% to 90.10% and 80.96% on the internal and external test sets, respectively.

**Table S2** Ablation experiments of CopFSNet model on the segmentation task

| FDA | DGA | FEP | Pruning | Dice | mIoU | GAcc. | HD95 |
| --- | --- | --- | --- | --- | --- | --- | --- |
| Internal test | | |  |  |  |  |  |
| × | × | × | – | 0.7702 | 0.8215 | 0.9495 | 15.96 |
| × | √ | × | – | 0.7838 | 0.8364 | 0.9684 | 15.12 |
| √ | × | √ | √ | 0.7926 | 0.8410 | 0.9724 | 13.72 |
| √ | √ | √ | × | 0.8003 | 0.8472 | 0.9768 | 13.21 |
| √ | √ | √ | √ | **0.8067** | **0.8518** | **0.9812** | **12.86** |
| External test | | |  |  |  |  |  |
| × | × | × | – | 0.7413 | 0.8103 | 0.9501 | 19.76 |
| × | √ | × | – | 0.7520 | 0.8164 | 0.9521 | 17.92 |
| √ | × | √ | √ | 0.7682 | 0.8285 | 0.9562 | 17.38 |
| √ | √ | √ | × | 0.7714 | 0.8327 | 0.9579 | 16.42 |
| √ | √ | √ | √ | **0.7812** | **0.8403** | **0.9604** | **15.64** |

NOTE: “mIoU” refers to the Mean Intersection over Union. “Gacc.” Denotes the global pixel-level segmentation accuracy. “HD95” represents the 95^th^ percentile of the Hausdorff Distance.

Table S4 summarizes the ablation results for the patient-level classification task, where all three modules and the pruning operation likewise enhanced performance, increasing accuracy from 83.42% and 71.82% to 91.43% and 85.95% on the internal and external test sets, respectively. Notably, removing feature sharing resulted in a substantial decline in classification performance in both Table S3 and Table S4, underscoring that leveraging segmentation features, which contain fine grained structural information, effectively supports and strengthens the image classification task.

**Table S3** Ablation experiments of CopFSNet model on the classification task

| FDA | DGA | FEP | Pruning | Feature sharing | Acc. | Precision | Recall | F1-score | AUC |
| --- | --- | --- | --- | --- | --- | --- | --- | --- | --- |
| Internal test | | |  |  |  |  |  |  |  |
| × | × | × | – | √ | 0.8575 | 0.8047 | 0.7832 | 0.7926 | 0.9053 |
| × | √ | × | – | √ | 0.8657 | 0.8283 | 0.7796 | 0.7982 | 0.9273 |
| √ | × | √ | √ | √ | 0.8823 | 0.8315 | 0.8249 | 0.8315 | 0.9452 |
| √ | √ | √ | × | √ | 0.8891 | 0.8427 | 0.8361 | 0.8429 | 0.9488 |
| – | – | – | – | × | 0.8232 | 0.7594 | 0.7395 | 0.7404 | 0.8794 |
| √ | √ | √ | √ | √ | **0.9010** | **0.8592** | **0.8555** | **0.8573** | **0.9572** |
| External test | | |  |  |  |  |  |  |  |
| × | × | × | – | √ | 0.7428 | 0.7282 | 0.6952 | 0.7136 | 0.8162 |
| × | √ | × | – | √ | 0.7661 | 0.7638 | 0.7104 | 0.7351 | 0.8427 |
| √ | × | √ | √ | √ | 0.7924 | 0.7882 | 0.7289 | 0.7628 | 0.8688 |
| √ | √ | √ | × | √ | 0.8018 | 0.7951 | 0.7297 | 0.7711 | 0.8715 |
| – | – | – | – | × | 0.7265 | 0.7187 | 0.6893 | 0.7037 | 0.7823 |
| √ | √ | √ | √ | √ | **0.8096** | **0.8284** | **0.7321** | **0.7773** | **0.8804** |

NOTE: “Acc.” Represents Accuracy. “AUC” represents the area under the AUC curve.

**Table S4** Ablation experiments of CopFSNet model on the patient-level classification task. This table shows the classification accuracy, and the average deviation between the predicted probability values and the true values

| FDA | DGA | FEP | Pruning | Feature sharing | Internal test | | External test | |
| --- | --- | --- | --- | --- | --- | --- | --- | --- |
|  |  |  |  |  | Accuracy | Deviation | Accuracy | Deviation |
| × | × | × | – | √ | 0.8342 | 0.2852 | 0.7182 | 0.2964 |
| × | √ | × | – | √ | 0.8568 | 0.2415 | 0.7568 | 0.2659 |
| √ | × | √ | √ | √ | 0.8821 | 0.1754 | 0.8204 | 0.2162 |
| √ | √ | √ | × | √ | 0.8986 | 0.1625 | 0.8364 | 0.2108 |
| – | – | – | – | × | 0.8211 | 0.3008 | 0.6872 | 0.3316 |
| √ | √ | √ | √ | √ | **0.9143** | **0.1567** | **0.8595** | **0.1965** |

**Reference**

1. Yao T, Pan Y, Li Y, Ngo CW, Mei T. Wave-vit: unifying wavelet and transformers for visual representation learning. In: European conference on computer vision (ECCV). 2022;328-345.
2. Rao Y, Zhao W, Zhu Z, Lu J, Zhou J. Global filter networks for image classification. In: Advances in neural information processing systems (NeurIPS). 2021;34:980-993.
3. Dosovitskiy A, Beyer L, Kolesnikov A, et al. An image is worth 16x16 words: Transformers for image recognition at scale, In: International conference on learning representations (ICLR). 2021;1-21.
4. Patro B, Agneeswaran V. Scattering vision transformer: spectral mixing matters. In: Advances in neural information processing systems (NeurIPS). 2024;36:1-15.
5. Han K, Wang Y, Tian Q, et al. Ghostnet: more features from cheap operations. In: Proceedings of the IEEE conference on computer vision and pattern recognition (CVPR). 2020;1580-1589.
6. Wu H, Huang X, Guo X, Wen Z, Qin J. Cross-image dependency modeling for breast ultrasound segmentation. IEEE Transactions on Medical Imaging. 2023;42(6):1619-1631.
7. Xia Z, Pan X, Song S, et al. Vision transformer with deformable attention. In: Proceedings of the IEEE conference on computer vision and pattern recognition (CVPR). 2022;4794-4803.
8. Chen Z, Badrinarayanan V, Lee C Y, et al. Gradnorm: gradient normalization for adaptive loss balancing in deep multitask networks. In: International conference on machine learning (ICML). 2018;794-803.
